# Influence of Apelin-13 on osteoporosis in Type-2 diabetes mellitus: A clinical study

**DOI:** 10.12669/pjms.341.14135

**Published:** 2018

**Authors:** Supin Liu, Wenlong Wang, Linlin Yin, Yitang Zhu

**Affiliations:** 1 Supin Liu, Blood Collection Centre, The Centre Hospital of Cangzhou, Cangzhou City, Hebei Province, China; 2 Wenlong Wang, Department of Clinical Laboratory, The Centre Hospital of Cangzhou, Cangzhou City, Hebei Province, China; 3 Linlin Yin, Department of Clinical Laboratory, The Centre Hospital of Cangzhou, Cangzhou City, Hebei Province, China; 4 Yitang Zhu, Department of Clinical Laboratory, The Centre Hospital of Cangzhou, Cangzhou City, Hebei Province, China

**Keywords:** Apelin-13, Bone mineral density (BMD), Osteoporosis, Type-2 diabetes mellitus

## Abstract

**Objective:**

To investigate the relationship between serum level of Apelin-13 and bone mineral density (BMD) as well as other parameters, and determine the influence of Apelin-13 on osteoporosis in patients with Type-2 diabetes mellitus.

**Methods:**

Seventy-six patients with Type-2 diabetes mellitus were recruited from Department of Endocrinology of our hospital between January 2013 and July2017. The clinical data, including age, gender, height, weight, body mass index (BMI) and disease duration were recorded for all patients. Blood sample was collected for measurement of Apelin-13, Procollagen type-I N propeptide (PINP) and Cross-linked carboxy terminal telopeptide of type-I collagen (ICTP), and BMD was tested with a dual-energy X-ray absorptiometry scanner.

**Results:**

The patients were divided into three groups, in which 19 patients were assigned in osteoporosis group, 25 in osteopenia group and 32 in normal group. The level of Apelin-13 in osteoporosis group was significantly lower than that in osteopenia and normal groups (p<0.05), and the value in osteopenia group was significant lower than that in normal group (p<0.05). Correlation analysis showed in the included patients the level of Apelin-13 was positively correlated to the value of BMD and PINP (p<0.05), but negatively correlated to age and ICTP (p<0.05).

**Conclusion:**

In conclusion, this study demonstrated that there was a close relationship among Apelin-13, BMD, ICTP and PINP, and Apelin-13 plays an important role in the occurrence of osteoporosis in patients with Type-2 diabetes mellitus.

## INTRODUCTION

Diabetes is one of the most challenging health problems in the world and its incidence has rapidly increased in recent years.^1^,^2^ The disease is closely correlated to fragile fractures resulted from osteoporosis, and Type-2 diabetes mellitus has been identified as an important risk factor for osteoporosis-associated fracture.^3^ The mechanism for bone fragility in diabetes mellitus is complicated, which may include hyperglycaemia, oxidative stress, advanced glycation end products, treatment-induced hypoglycaemia, certain anti-diabetic medications with a direct effect on bone and mineral metabolism as well as an increased propensity for falls, all contribute to the increased fracture risk in patients with Type-2 diabetes mellitus.^4^ The association between diabetes-related osteoporosis and mortality in Type-2 diabetes mellitus has also been confirmed.^5^ However, some patients with Type-2 diabetes mellitus present lower bone mineral density (BMD), some present normal or increased BMD,^6^ this indicates some factors may affect the level of BMD in patients with Type-2 diabetes mellitus, and detection of these factors and their associations may facilitate physicians in preventing osteoporosis in patients with Type-2 diabetes mellitus, but few studies have been carried out in this regard.

Moreover, Apelin is a peptide and endogenous ligand of human G-protein. Many studies demonstrate that Apelin can regulate glucose homeostasis, insulin secretion and sensitivity.^7^ *Apelin-13* is one of the most studied types of apelin, which has emerged as a beneficial peptide with anti-obesity and anti-diabetic properties, and is regarded as a promising therapeutic target in metabolic disorders.^8^ Some studies suggest that *Apelin-13* can regulate multiple physiological functions and is closely associated with diabetes, obesity, hypertension and cardiovascular diseases.^1^ In a study of sixty-nine patients with Type-2 diabetes mellitus, Du and colleagues found that serum level of Apelin-13 was significantly elevated in patients with proliferative diabetic retinopathy, suggesting a positive association of Apelin-13 with proliferative diabetic retinopathy.^9^ Diabetic nephropathy is the primary cause of end-stage renal disease and in a recent published study, Chen advocates that Apelin-13 may be a novel therapeutic candidate for it via regulation of histone acetylation.^10^ In addition, Apelin-13 also can alleviate diabetes-associated endoplasmic reticulum stress in the pancreas.^11^ These studies demonstrate that Apelin-13 play an important role in the treatment of diabetes related complications. Subsequently, we hypothesized that the serum level of Apelin-13 may be associated with the occurrence of osteoporosis in patients with Type-2 diabetes mellitus. However, up to now, the relationship between Apelin-13 and BMD has not yet been investigated.

Therefore, the purpose of this study was to investigate the relationship between the serum level of Apelin-13 and BMD as well as other parameters, and determine the influence of Apelin-13 on osteoporosis in patients with Type-2 diabetes mellitus.

## METHODS

Seventy-six patients with Type-2 diabetes mellitus were recruited from Department of Endocrinology during January 2013 to July 2017. The clinical data including age, gender, height, weight, body mass index (BMI) and disease duration were recorded for all patients. The inclusion criteria of current study were (1) patients diagnosed with Type-2 diabetes mellitus and (2) those who agreed to participate this study and signed a written informed consent for all examinations and procedures at the beginning of the study, but those with blood disease, malignant tumors, thyroid disease, parathyroid disease, autoimmune diseases, inflammatory diseases and severe heart, liver and kidney disease were excluded. This study was approved by Ethics Committee of our hospital, all procedures were performed in accordance with ethical approval institutional guidelines.

After overnight fasting, blood sample was collected for measurement of Apelin-13, Procollagen type-I N propeptide (PINP) and Cross-linked carboxy terminal telopeptide of type-I collagen (ICTP) on the second day after admission. Serum level of Apelin-13 were measured with commercial human enzyme linked immunosorbent assay kits, and the serum level of PINP and ICTP was measured with radioimmunoassay method. In addition, BMD was tested with a dual-energy X-ray absorptiometry scanner by two experienced technicians. The measurement of BMD in each subject was performed at left hip and lumbar spine from L1 to L4. To evaluate the level of BMD, T-scores were used and classified based on the World Health Organization criteria: T-score ≤−2.5 SD indicates osteoporosis; T-score >−2.5 and <−1.0 SD indicates osteopenia; T-score ≥−1.0 indicates normal.^3^ The included patients were divided into normal, osteopenia and osteoporosis group according to the outcome of BMD measurements.

SPSS version 21.0 (SPSS Inc., Chicago, IL, USA) was used for statistical analysis and p < 0.05 was considered significant. Independent two-sample t-test or Analysis of variance was performed to compare the difference of measurement data, and a chi-square test was employed to compare the difference of enumeration data between two groups. Correlation analyses was performed by Pearson's correlation analysis.

**Fig. 1:**
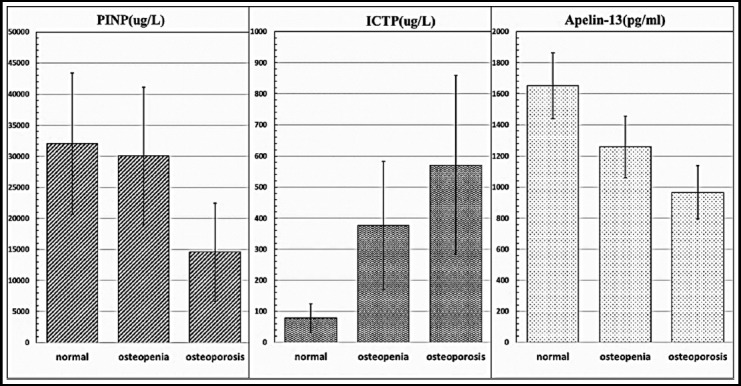
The comparison of PINP, ICTP and Apelin-13 in normal, osteopenia and osteoporosis groups.

## RESULTS

In the current study, a total of 76 patients with Type-2 diabetes mellitus were included. They were divided into three groups, in which 19 patients were assigned in osteoporosis group, 25 in osteopenia and 32 in normal group based on T scores outcomes. The rate of osteoporosis, osteopenia and normal was 25%, 32.9% and 42.1% respectively in three groups.

The basic clinical data including age, gender, weight, body mass index (BMI), height, and disease duration, Level of Apelin-13, PINP and ICTP are listed in Table-I. There were significant differences in age, gender, level of Apelin-13 and ICTP (p<0.05) among the three groups. The level of PINP was significantly higher in normal and osteopenia group than osteoporosis group (p<0.05), the value in normal group was also higher than that in osteopenia group, but no significant difference was found (p>0.05). The level of ICTP was significantly lower in normal and osteopenia group than osteoporosis group (p<0.05), and the value in normal group was also lower than that in osteopenia group (p<0.05). The level of Apelin-13 in osteoporosis group was significantly lower than that in osteopenia and normal groups (p<0.05), and the value in osteopenia group was also significantly lower than that in normal group (p<0.05). In addition, there were no significant differences in BMI, height, weight and disease duration among the three groups (p>0.05, Table-I).

**Table-I:** General characteristics of patients in the three groups.

	Normal	Osteopenia	Osteoporosis	P-value
Number	32	25	19	-
Gender(F/M)	18/14	7/18	12/7	0.037
Age (year)	53±11	56±12	61±13	0.02
Weight (Kg)	77±21	79±19	81±23	0.08
Height (cm)	171±15	169±16	170±13	0.82
BMI	27.3±3.0	28.1±2.9	26.5±3.1	0.51
Disease duration(year)	4.4±3.8	5.4±4.7	7.6±6.2	0.79
Apelin-13 (Pg/ml)	1651±213	1258±198	967±172	0.01
PINP (ug/L)	32019±11369	30075±10067	14551±7891	0.03
ICTP (ug/L)	78.9±45.4	376.9±206.7	571.6±287.9	0.002

Moreover, correlation analysis showed that in the included patients the level of Apelin-13 was positively correlated to the value of BMD and PINP (p<0.05), but negatively correlated to age and ICTP (p<0.05).

## DISCUSSION

In this study, we investigated the relationship between Apelin-13 level and BMD as well as other parameters to determine the influence of Apelin-13 on osteoporosis in patients with Type-2 diabetes mellitus, which may provide some clinical evidences for physicians in preventing osteoporosis. To the best of our knowledge, few studies have been carried out in this field.

It is apparent that patients with Type-2 diabetes mellitus are at high risk of fracture. However, some studies indicated that patients presented generally normal or increased BMD^6^. Although some other factors, such as diabetic complications, may lead to increased risk of fall and fracture in these patients, the main reason may be attributed to the value of BMD.^12^ In the current study, we found the level of Apelin-13 in osteoporosis and osteopenia group was significantly lower than that in normal group, and multivariate analysis showed that in all included patients the level of Apelin-13 was positively correlated to the value of BMD, demonstrating that Apelin-13 may be a protective factor for osteoporosis in patients with Type-2 diabetes mellitus.

In addition, PINP, an osteoblast-derived protein, is one biological response marker during treatment for osteoporosis.^13^ At the same time, ICTP, as a specific component of type I collagen, generates from damaged mature bone matrix, and can represent a sensitive indicator of bone resorption in vivo.^14^ Subsequently, in the current study we evaluate the relation between Apelin-13, ICTP and PINP to detect the influence of Apelin-13 on osteoporosis. We found a close relationship between Apelin-13, PINP and ICTP; the level of Apelin-13 was positively correlated to PINP, but negatively correlated to ICTP. This demonstrated that Apelin-13 may be a biological marker in supervising bone resorption or formation.

Some authors studied the relation between osteoporosis and Type-2 diabetes mellitus and related risk factors for osteoporosis. In a study of forty patients with Type-2 diabetes mellitus, Zhang and colleagues found that the prevalence of osteoporosis is closely related with gender, BMI, disease course and glucose level.^15^ In another study of 87 patients with Type-2 diabetes mellitus, Wang found that between normal control group and osteoporosis group, there were significant differences in gender, age, weight and height, but no differences in BMI and disease course.^16^ In the current study, we found there were significant differences in age and gender, but not in disease course, BMI, weight, and height among the three groups. In terms of the risk factors related to osteoporosis in Type-2 diabetes mellitus, some viewpoints are similar, but some are different, which may be attributed to many factors such as sample size and patient selection. However, most of the studies demonstrated that gender is correlated to the occurrence of osteoporosis in Type-2 diabetes. The difference in BMD distribution between genders may be explained by the differences in endocrine and paracrine factors, and the rapid decrease of sex steroid levels in postmenopausal women aggravates the bone loss.^17^ In addition, we found that the level of Apelin-13 was negatively related to age, demonstrating the risk of osteoporosis may be added with age, this conclusion was consistent with previous studies.^17^

In conclusion, our study demonstrated that there was a close relationship between Apelin-13, BMD, ICTP and PINP, suggesting that Apelin-13 plays an important role in the occurrence of osteoporosis in patients with Type-2 diabetes mellitus. However, the current study has its limitations. First, the close relationship between Apelin-13, BMD, ICTP and PINP were found, but their interaction and related mechanism were still unclear. Second, in this study only patients with Type-2 diabetes mellitus were included and no normal controls were recruited, but the level of Apelin-13 as well as abovementioned relations in normal populations may be different. Third, the sample size was small and some comparisons showed no significance, in a study with large scale sample the results may be different. Thus, all these issues need to be studied and resolved in future.

### Author`s Contribution

**SPL** conceived, designed and did statistical analysis & editing of manuscript.

**SPL, WLW, LLY & YTZ** did data collection and manuscript writing.

**SPL** did review and final approval of manuscript.
